# Deep Learning-Based Automated Approach for Determination of Pig Carcass Traits

**DOI:** 10.3390/ani14162421

**Published:** 2024-08-21

**Authors:** Jiacheng Wei, Yan Wu, Xi Tang, Jinxiu Liu, Yani Huang, Zhenfang Wu, Xinyun Li, Zhiyan Zhang

**Affiliations:** 1 National Key Laboratory of Swine Genetic Improvement and Germplasm Innovation, Jiangxi Agricultural University, Nanchang 330045, China; 2 College of Animal Science and National Engineering Research Center for Breeding Swine Industry, South China Agricultural University, Guangzhou 510642, China; 3 Key Laboratory of Swine Genetics and Breeding, Ministry of Agriculture, Huazhong Agricultural University, Wuhan 430070, China

**Keywords:** deep learning, automated phenotyping of pigs, carcass length, backfat thickness

## Abstract

**Simple Summary:**

Utilizing computer vision technology to automate the measurement of pig carcass traits is of significant importance for genetic selection in breeding and enhancing the economic efficiency. However, currently, there is limited research, both domestically and internationally, on the measurement of pig carcass traits. This study proposes an automated system for measuring carcass traits using deep learning methods. The system aims to standardize and accelerate the acquisition of carcass traits from large groups of subjects. This approach seeks to uncover numerous micro-effect variant loci that have not yet been explored through large-sample GWAS analyses.

**Abstract:**

Pig carcass traits are among the most economically significant characteristics and are crucial for genetic selection in breeding and enhancing the economic efficiency. Standardized and automated carcass phenotyping can greatly enhance the measurement efficiency and accuracy, thereby facilitating the selection and breeding of superior pig carcasses. In this study, we utilized phenotypic images and data from 3912 pigs to propose a deep learning-based approach for the automated determination of pig carcass phenotypic traits. Using the YOLOv8 algorithm, our carcass length determination model achieves an average accuracy of 99% on the test set. Additionally, our backfat segmentation model, YOLOV8n-seg, demonstrates robust segmentation performance, with a Mean IoU of 89.10. An analysis of the data distribution comparing manual and model-derived measurements revealed that differences in the carcass straight length are primarily concentrated between −2 cm and 4 cm, while differences in the carcass diagonal length are concentrated between −3 cm and 2 cm. To validate the method, we compared model measurements with manually obtained data, achieving coefficients of determination (R^2^) of 0.9164 for the carcass straight length, 0.9325 for the carcass diagonal length, and 0.7137 for the backfat thickness, indicating high reliability. Our findings provide valuable insights into automating carcass phenotype determination and grading in pig production.

## 1. Introduction

In modern pig farming, carcass traits play a pivotal role in selecting the breeding stock and optimizing precision feeding strategies. Among these traits, backfat thickness is particularly crucial in determining the reproductive performance of sows. It significantly influences outcomes such as puberty attainment, the total number of piglets born, and the farrowing rate, as it is linked to key hormones like leptin, insulin-like growth factor-I (IGF-I), and progesterone (P4) [1]. Studies have highlighted the profound impact of backfat thickness and its fluctuations throughout the reproductive cycle on the reproductive performance of sows [2]. Additionally, backfat thickness has been strongly associated with various meat quality traits, underscoring its multifaceted importance in pig farming [3]. Given its significance, the rapid and accurate measurement of backfat thickness is essential for efficient genetic selection in breeding and enhancing the economic efficiency [4,5,6]. Carcass length is another significant trait that breeders focus on to improve the pork yield by selecting pigs with larger body sizes. While numerous genes associated with carcass length have been identified through genetic studies [7,8,9], these findings only explain a portion of the heritability, leaving considerable genetic variance unexplained [10]. Genome-wide association studies (GWASs) of large samples can identify numerous micro-efficient variant loci and represent a crucial strategy in understanding the heritability of traits [11,12,13]. However, manually conducting extensive phenotypic assays is time consuming, inefficient, and prone to subjective measurement errors among different assessors [14,15,16]. Hence, there is a pressing need for standardized, rapid, and automated methods to gather carcass trait data from large populations, which would enhance the measurement consistency and scalability in breeding programs.

Carcass characterization plays a pivotal role in assessing the quality and value of slaughtered animals [17]. Classification systems categorize carcasses based on specific descriptors, while grading aims to assess carcass merit, incorporating an element of value [18]. Carcass evaluation has become essential for ensuring quality control in commercial transactions between livestock producers and meat processors. A deeper understanding of carcass quality and composition enables a more precise allocation of meat cuts according to market demands [17]. Criterion-based subjective evaluation serves as the foundation for carcass classification. However, to enhance their consistency and accuracy, various objective techniques have been integrated into carcass assessment systems [17]. Non-destructive methods, such as Computed Tomography (CT), Magnetic Resonance Imaging (MRI), and ultrasound, have shown the potential to complement or replace traditional approaches [19]. Emerging technologies like computer vision and hyperspectral imaging are promising advancements in this field. Nevertheless, the high initial cost of hyperspectral imaging systems currently limits their widespread adoption [20]. Given the complexity of carcass images, effective image segmentation algorithms represent one of the most challenging tasks [21]. Achieving reliable and consistent segmentation of carcass images, without human intervention, is crucial for subsequent automated grading processes [22].

With the continuous advancement of computer vision technology, Convolutional Neural Networks (CNNs) have become the cornerstone of modern computer vision methods, offering high precision and accuracy across various agricultural applications [23]. Compared to conventional image processing methods, deep learning-based computer vision can automatically perform various agricultural activities with the highest accuracy, making smart agriculture a reality [24]. In agricultural target detection tasks, an increasing number of deep learning models are being employed to meet diverse requirements in agricultural phenotyping. Examples include YOLOv5, YOLOv8, Faster R-CNN, SSD, and others [25,26,27,28]. In agricultural target segmentation tasks, the landscape is evolving with the introduction of advanced deep learning models known for their robust performance. Models such as U-Net, DeepLab, and PSPNet have gained prominence [29,30,31,32,33,34]. With the advent of Convolutional Neural Networks, research on the automatic determination of pig carcass traits based on deep learning has ushered in great opportunities with significant potential for application. Lee et al. proposed a deep learning method, BTENet, for backfat thickness estimation of commercial pigs in Korea, and their experimental results verified that BTENet achieves reliable thickness estimations (Pearson’s correlation coefficient: 0.915; mean absolute error: 1.275 mm; mean absolute percentage error: 6.4%) [35]. However, there are few studies on methods for the automatic determination of phenotypic traits in pig carcasses using deep learning methods.

Therefore, in this study, we proposed an automated method for the determination of phenotypic carcass traits by incorporating a deep learning approach, with the aim of facilitating breeders to obtain a large number of carcass phenotypes in a short period of time and to obtain carcass traits from subjects with extreme pig body sizes, thus advancing the selection process.

## 2. Experimental Materials and Methods

### 2.1. Experimental Materials

In this study, we positioned the left half of the pig carcass obtained after slaughter in a fixed position and placed a 6 cm × 6 cm square iron sheet at the carcass’s center as an internal reference. This setup facilitated the subsequent calculations of the pig carcass length based on pixel points. Images were captured using a Xiaomi 10 smartphone (Xiaomi Corporation, a prominent technology company headquartered in Beijing, China; 4344 px × 5792 px) positioned 1 m away from the carcass. We ensured the image was centered on the carcass and aimed towards the front of its left half to capture a complete image, including the backfat. This approach avoided top-down and bottom-up camera angles and prevented one side of the carcass from biasing towards the camera, which could affect the visual accuracy.

This group of pigs is an experimental population designed by the National Key Laboratory of Pig Genetic Improvement and Innovation at Jiangxi Agricultural University. It includes three breeds—Duroc, Landrace, and Large White—totaling 3912 pigs. They are intended for studying and analyzing the genetic mechanisms underlying complex traits in pigs. We collected images of a total of 3912 pig carcasses, and during the image collection process, 501 were randomly selected for carcass length measurements using a tape measure for our subsequent comparative analysis. Similarly, images of the complete backfat of 777 pigs were acquired, with 159 subjects randomly selected to measure the fat thickness of the shoulder, loin, rump, 6th–7th rib, and back using a vernier caliper during the image acquisition process for subsequent studies.

### 2.2. Data Processing

**Figure 1:**
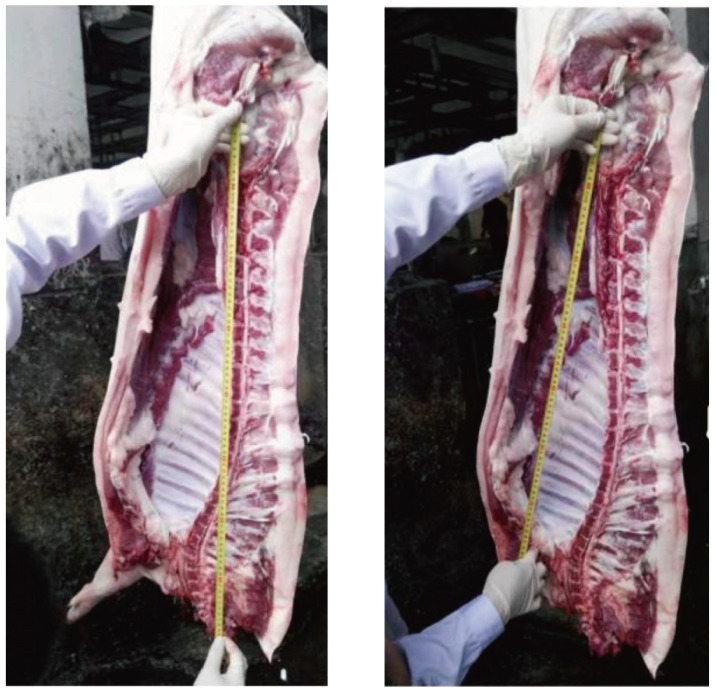
Example of carcass straight length and carcass oblique length.

Carcass length refers to both the oblique and straight lengths of the carcass, measured in centimeters. According to the Agricultural Industry Standard of the People’s Republic of China (NY/T 825-2004, Technical regulation for testing of carcasses traits in lean-type pig) [36], as depicted in Figure 1, during slaughter measurement, the left half of the carcass is hung upside down. A tape measure is used to measure the straight-line distance from the anterior edge of the pubic symphysis to the inner edge where the first rib meets the sternum, known as the carcass oblique length. The straight-line distance from the anterior edge of the pubic symphysis to the front edge of the first cervical vertebra is known as the carcass straight length. Therefore, we used LabelImg software (V1.8.6) to annotate the 3912 images we collected, with the annotations including categories such as the pubic symphysis, the first rib, the first cervical vertebra, and internal references. To assess the model’s accuracy, we divided the annotated images of 3411 pigs into training and validation sets at an 8:2 ratio for model training and validation. We used the annotated images of carcasses from 501 randomly selected pigs collected during the image acquisition process as the test set to evaluate the model’s performance. Finally, we evaluated the trained model by comparing its automatic measurements of the carcass straight length and carcass oblique length in the test set with those obtained manually as the gold standard.

Similarly, Labelme software was used to annotate the complete backfat and internal parameters in 777 images. After completing the annotation, the training set was converted into formats required by the deep learning model, such as VOC or YOLO format. To assess the model’s accuracy, we divided the dataset, consisting of annotated images of complete backfat from 618 individual pigs, into training and validation sets at an 8:2 ratio for model training and validation. Complete backfat images of 159 pigs, randomly selected during the image acquisition process, were assigned as the test set to evaluate the model’s performance. Finally, we evaluated the trained model by comparing the average backfat thickness automatically determined by the model in the test set with the five-point average backfat thickness obtained from the gold standard manual measurements.

Here, we need to emphasize that since we are creating datasets for two different tasks, their sizes are different, with 3912 and 777 samples, respectively. Regarding the number of subjects, which are 501 and 159, this decision was based on the available manually measured phenotype data. Therefore, we used images from 501 and 159 subjects as the testing set, with their manually measured data used for the subsequent comparative analysis. The remaining images are split into training and validation sets at an 8:2 ratio.

### 2.3. Construction of Determination Models

YOLOv8, the eighth iteration in the YOLO family, utilizes CSPDarknet53 as its backbone, features a decoupled head, and employs a path aggregation network in its neck for a lightweight yet accurate design. This approach aligns with advanced detection framework principles, ensuring stable and efficient training strategies that accelerate model convergence and enhance target detection performance. YOLOv8 supports non-YOLO models and various tasks such as classification, segmentation, and pose estimation, positioning it as one of the most advanced models available [37,38]. Faster R-CNN, an enhanced two-stage target detection algorithm, has found widespread application in tasks such as human pose recognition and target tracking [39]. SSD, a classical one-stage fast target detection model, combines the regression concepts of YOLO with the anchor box mechanism of Faster R-CNN, striking a balance between detection accuracy and speed [40]. Fully Convolutional Networks (FCNs) were introduced in 2015, pioneering the field of deep learning for semantic segmentation of images. FCNs replace the conventional fully connected layers at the end of CNNs with convolutional layers, transforming the network’s output into heatmaps rather than discrete categories. To counteract the spatial resolution reduction caused by convolution and pooling operations, FCNs utilize up-sampling techniques to restore the dimensions of the image [41]. U-Net adopts a U-shaped encoder–decoder architecture enriched with skip connections, facilitating the accurate segmentation of input images into distinct regions. The encoder employs convolution to progressively reduce the feature map resolution while capturing high-level semantic details. In the decoder, up-sampling and transposed convolutions restore feature maps to their original dimensions, refining predictions with the aid of skip connections for precise pixel-level segmentation. U-Net excels in generalization and resilience, finding application in medical imaging and nature image processing, among other fields [42]. DeepLab integrates Deep Convolutional Neural Networks (DCNNs) and Conditional Random Fields (CRFs) to enhance semantic image segmentation accuracy. DCNNs extract high-level features and semantic information, while CRFs ensure spatial consistency in predictions. DeepLab introduces Atrous convolution and Atrous spatial pyramid pooling (ASPP) to enrich DCNNs with contextual information across various scales within images, achieving efficient and detailed semantic segmentation [43]. PSPNet introduces a pyramid pooling module to address challenges in global scene context utilization. By integrating multi-scale information from different sub-regions, PSPNet constructs comprehensive global scene priors in deep neural network feature maps. This approach minimizes contextual information loss between sub-regions, thereby enhancing semantic segmentation performance [44].

In this study, we employed 10 deep learning algorithms to construct models for different carcass trait determinations and compared their performances. Four target detection algorithms were utilized to develop a carcass length determination model for automatically determining the straight and oblique carcass lengths, while six target segmentation algorithms were used to construct a backfat thickness segmentation model for automatically determining the backfat thickness.

The operating system used for model training and testing was Windows 10. The versions of Python and PyTorch employed for deep learning were 3.9 and 2.0.0, respectively. The CPU and GPU used were Intel Core i7-13700K (Intel Corporation, Santa Clara, CA, USA) and Nvidia GeForce RTX 4070 Ti (Nvidia Corporation, Santa Clara, CA, USA). The CUDA (Compute Unified Device Architecture) and CUDA deep neural network libraries used were versions 11.8 and 8.7.0, respectively. During the training process, the group size and number of epochs were 32 and 200, respectively, with an image size of 640, and other parameters were set to default values.

### 2.4. Model Evaluation

Based on the characteristics of the two models, we used different metrics to evaluate the performances of the models:

Since our carcass length determination model is designed for target detection tasks, we evaluated its performance using common metrics for such models: Mean Average Precision (mAP), number of parameters, and Floating-Point Operations (FLOPs):

(1) $$mAP=\frac{\sum_{1}^{n}\int_{0}^{1}Precison(Recall)d(Recall)}{n}$$

where TP, FP, and FN are the number of true positives, false positives, and false negatives, respectively. In Equation (1), AP is the area under the precision–recall curve (P-R curve), and mAP is the mean of different categories of AP.

Similarly, since our backfat thickness determination model is based on a target segmentation task, we assessed its performance using the most commonly used evaluation metrics for target segmentation models. Five evaluation metrics were calculated using the confusion matrix between the mask image and the ground truth obtained after the model determination. Mean accuracy represents the average proportion of correctly predicted pixels among the predicted pixels for each class, as described in Equation (2). MIoU refers to the mean intersection on the union (MIoU), which is determined by calculating the ratio of the intersection area between the ground truth and predicted values for each class to the joint area. The mean intersection on the union (IoU) was determined by calculating the ratio of the ground truth values to the predicted values for each class and then averaging these values, as described in Equation (3). Recall is the ratio of correctly predicted pixels in the predicted backfat area, as shown in Equation (5). F1-Score is a metric for evaluating the accuracy of object boundaries. It is determined by calculating the precision and recall of the boundary pixels, as shown in Equation (6).

(2) $$Mean\;Accuracy=\frac{1}{2}(\frac{TN}{TN+FP}+\frac{TP}{TP+FN})$$

(3) $$Mean\;IoU=\frac{1}{2}(\frac{TP}{FN+TP+FP}+\frac{TN}{FN+TN+FP})$$

(4) $$Precision=\frac{TP}{TP+FP}$$

(5) $$Recall=\frac{TP}{TP+FN}$$

(6) $$F1-Score=\frac{2\times Precison\times Recall}{Precision+Recall}$$

True positives (TP) represent the number of pixels in the correctly predicted backfat region, false negatives (FN) represent the number of pixels in the incorrectly predicted background instead of the backfat region, false positives (FP) represent the number of pixels in the incorrectly predicted backfat region instead of the background, and true negatives (TN) represent the number of pixels in the correctly predicted background.

### 2.5. Statistical Analysis

This study evaluates our proposed method by collecting data on body lengths and backfat thicknesses utilizing linear regression analysis. We employed the ordinary least squares method to establish the linear model, and the goodness of fit of the linear regression model was assessed by the R^2^ value, quantifying the model’s explanatory power over the data. All statistical analyses were conducted using the Python library Scikit-Learn to ensure the reliability and scientific validity of the results.

## 3. Results and Analysis

### 3.1. Evaluation and Comparison of Model Performance

Table 1 presents the evaluation results of various target detection models on the test set. Apart from the Faster R-CNN model, the three other models across four detection classes—the pubis, first rib, first cervical vertebra, and internal reference—achieved Mean Average Precision values exceeding 95%. Notably, the YOLOv8 model demonstrated the highest detection performance, achieving Mean Average Precisions of 98.2%, 99.4%, 99.1%, and 99.5% for these respective categories. Despite the YOLOv8 model having 1.25 M more parameters and 3.9 G more FLOPs compared to the YOLOv5 model, this difference did not significantly affect practical applications. Moreover, the Mean Average Precision of the YOLOv8n model exceeded that of the YOLOv5n model by 1.9%, 1.5%, 0.1%, and 0.1% across the same four detection classes. Consequently, considering both detection precision and the model parameters, we selected the YOLOv8 model for practical applications in carcass length detection.

Table 2 presents the segmentation evaluation results of six deep learning models on the test set. Except for the FCN model, the remaining five deep learning models achieve Mean Accuracy, precision, recall, and F1-Score scores above 93%. The FCN model shows the poorest performance, with only 69.65 mIoU. In contrast, the YOLOv8-seg model demonstrates a superior segmentation performance, with a Mean Accuracy of 97.23, Mean IoU of 89.10, precision of 96.86, recall of 97.23, and F1-Score of 97.03. Based on these results, we selected the YOLOv8-seg model for practical applications in backfat region segmentation.

### 3.2. Results of Training the Carcass Length Determination Model and Comparative Analysis

**Figure 2:**
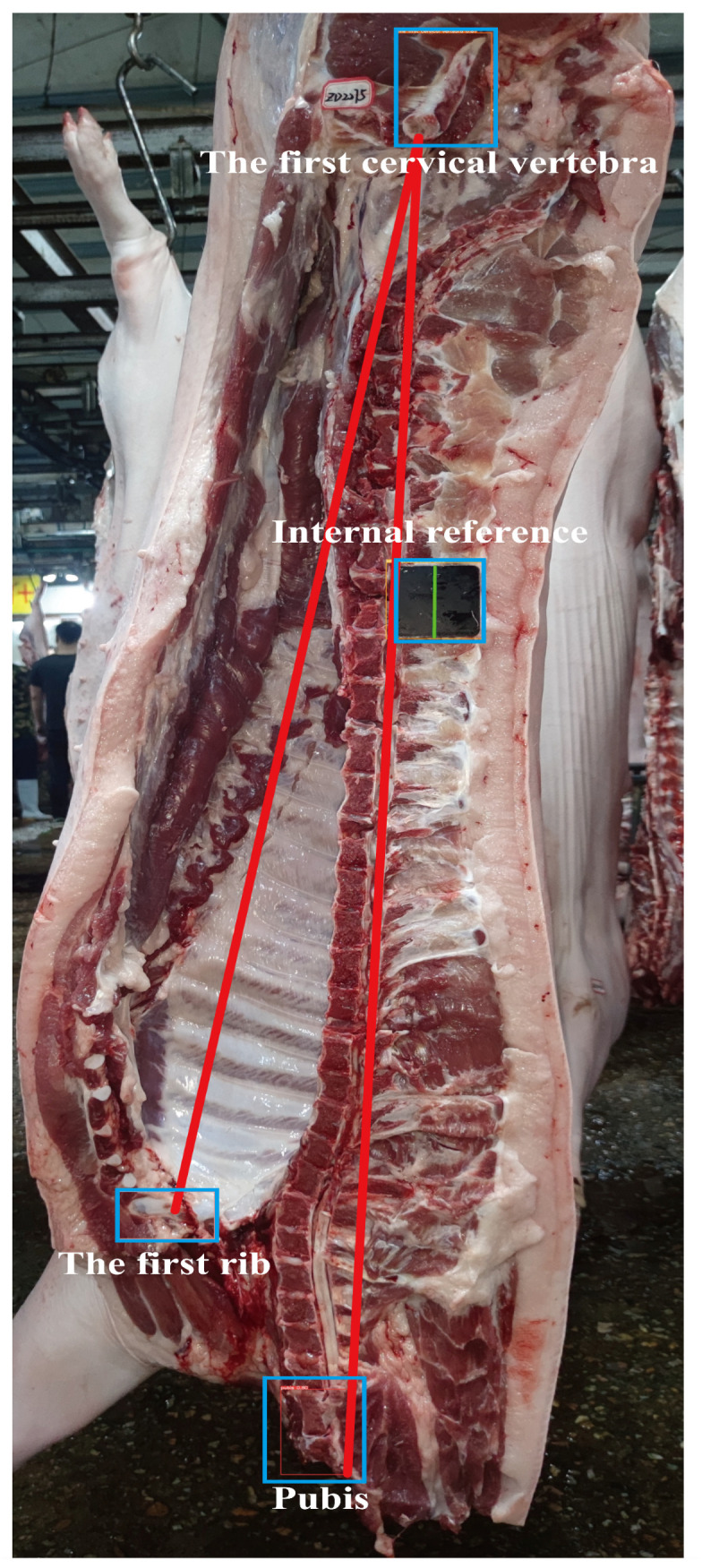
Example of a carcass length determination model.

**Figure 3:**
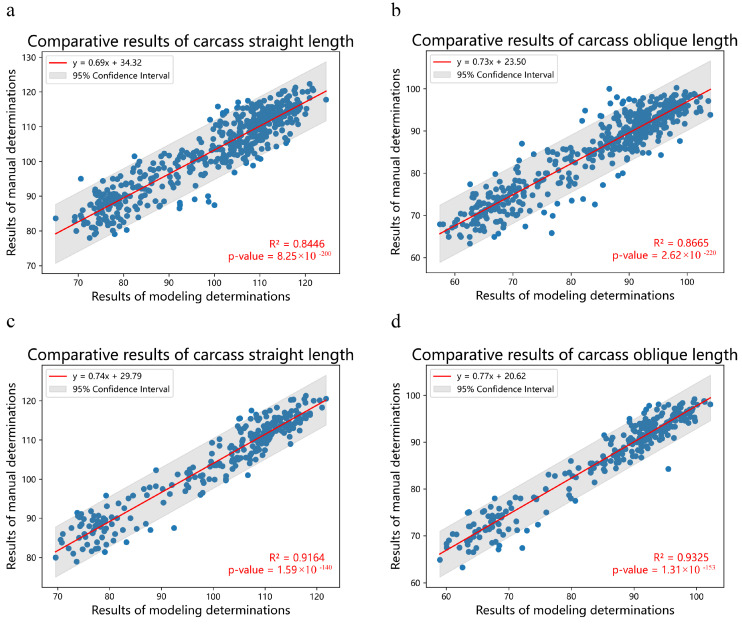
Comparative analysis of model determination and manual measurement of carcass length before and after image filtering. (**a**,**b**) Unfiltered carcass straight length and carcass slant length data; (**c**,**d**) filtered carcass straight length and carcass oblique length data.

Figure 2 illustrates an example of our model’s determination process, where our model accurately identified three landmarks: the anterior edge of the pubic symphysis, the junction of the inner edge of the first rib and sternum, and the anterior aspect of the first cervical vertebra, along with an internal reference. The coordinates of these four detection frames were extracted to calculate both the carcass straight length and the carcass oblique length. We evaluated the model by comparing the carcass straight length and carcass oblique length determined using model with those obtained manually. Figure 3 presents the results of this comparison. From Figure 3a,b, we observe that the coefficient of determination (R^2^) between the carcass straight length determined using the model and the manually measured carcass straight length is 0.8446. Similarly, the R^2^ between the carcass oblique length determined using the model and the manually measured carcass oblique length is 0.8665. Despite our efforts to minimize errors due to visual angle biases during image acquisition, we identified several outliers among the 501 images. Consequently, we meticulously reviewed and filtered these images. Notably, some images were captured from extreme angles, resulting in model measurements that were significantly larger or smaller than manual measurements. Figure 3c,d depict the results after filtering these error-prone images and comparing the model measurements with manual measurements. From these figures, we observe that the R^2^ between the carcass straight length obtained using model determination and the manual measurement increased from 0.8446 to 0.9164. Similarly, the R^2^ between the carcass oblique length obtained using model determination and the manual measurement increased from 0.8665 to 0.9325. These findings underscore the reliability of the automated carcass length detection method proposed in this study.

### 3.3. Comparative Analysis of Data Distribution before and after Image Filtering

**Figure 4:**
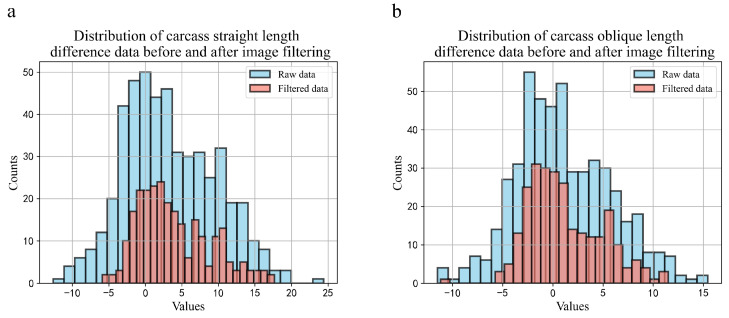
Data distribution of the differences in carcass lengths obtained using manual measurement and model determinations before and after image filtering. (**a**) Data distribution of the differences in carcass straight lengths before and after filtration; (**b**) data distribution of the differences in carcass oblique lengths before and after filtration.

We analyzed the differences in the carcass lengths obtained manually versus those predicted by our model before and after image filtering and examined their data distributions. Figure 4 illustrates the data distributions of the carcass straight length and carcass oblique length differences before image filtering. Initially, the carcass straight length differences were primarily distributed between −7 cm and +14 cm, with a maximum deviation of +24 cm and a minimum of −13 cm. Similarly, the carcass oblique length differences ranged from −6 cm to +9 cm, with a maximum deviation of +16 cm and a minimum of −12 cm. These findings are consistent with our previous observations, where varying image angles led to significant discrepancies between the model predictions and manual measurements. After filtering, the differences in the carcass straight length are primarily concentrated between −2 cm and +4 cm, with the data distribution skewed towards the right. We believe that during manual measurements with a tape measure, the pubic symphysis anterior edge and the first cervical vertebra notch are not on the same plane. However, when automatically capturing the carcass straight length from images, the pubic symphysis anterior edge and the first cervical vertebra notch are effectively aligned in the same plane, akin to measuring the hypotenuse of a triangle manually, whereas the model predicts the leg of the triangle. Therefore, the rightward skew in the distribution of differences aligns with practical expectations. Filtered carcass oblique length differences were primarily concentrated between −3 cm and +2 cm, with a left-skewed distribution. These results indicate an acceptable margin of error and underscore the reliability of our automated carcass length detection method.

### 3.4. Training Results of the Backfat Segmentation Model and Comparative Analysis

**Figure 5:**
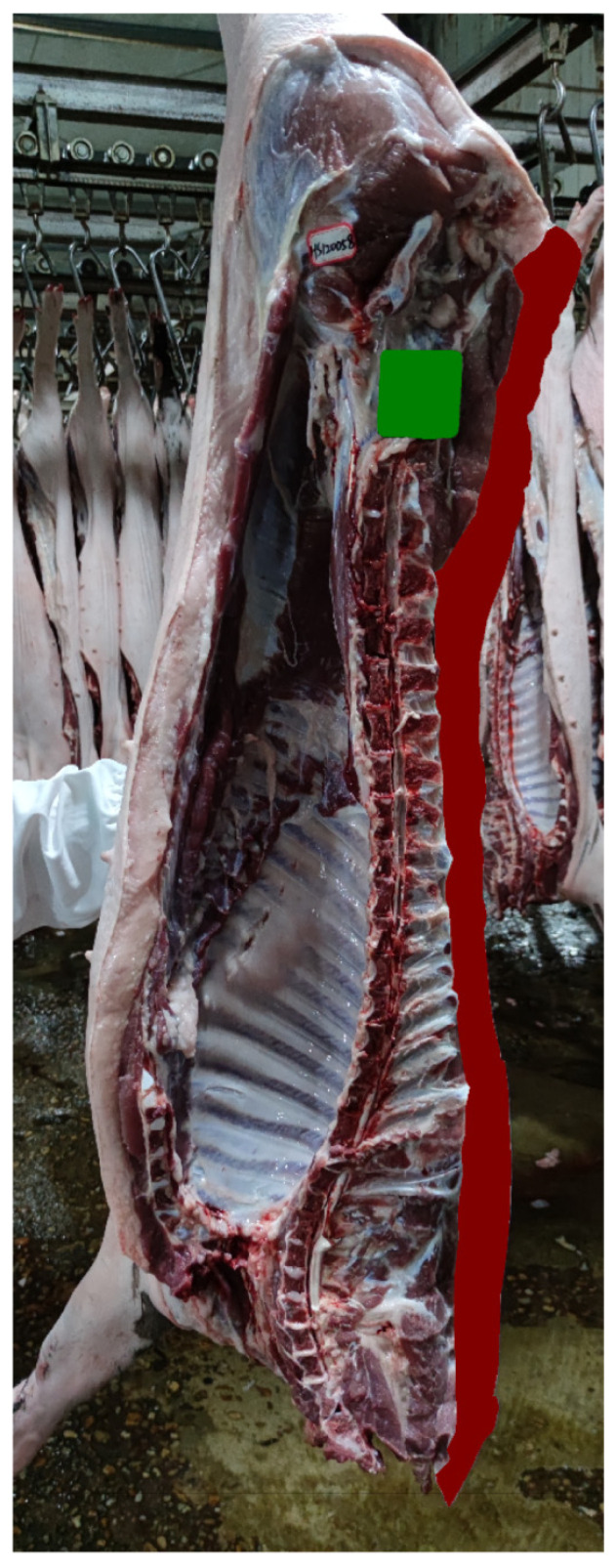
Example of segmentation results of the automatic backfat segmentation model.

**Figure 6:**
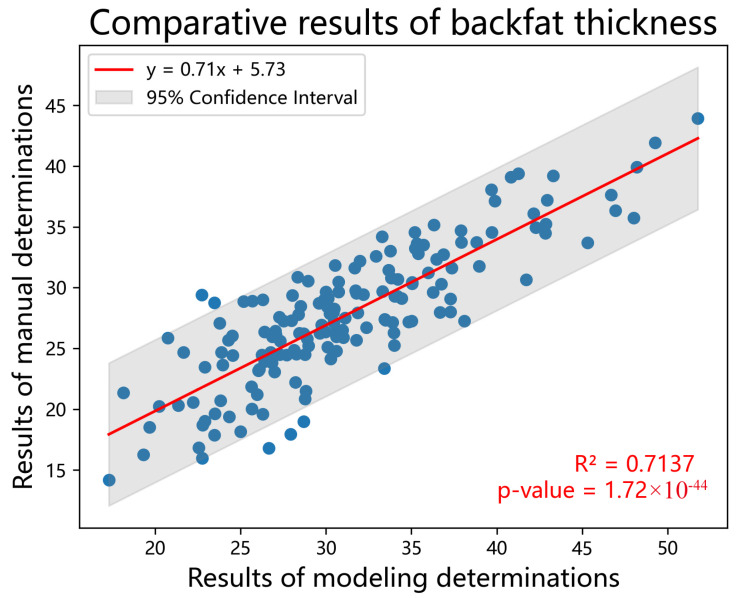
Results of the comparative analysis between the mean backfat thickness obtained using the model and the five-point mean fat thickness obtained by manual measurement.

The segmentation results of the automatic backfat segmentation model we developed are presented in Figure 5. From these results, it is evident that our model accurately segments the entire backfat of the carcass, including the internal reference. We divided the backfat area obtained using the segmentation model by the carcass straight length predicted by our model to derive an average backfat thickness. We evaluate the model by comparing the average backfat thickness obtained using the model with the average fat thickness measured manually at five points: the 6th–7th ribs, back, shoulder, loin, and rump. The comparison results are shown in Figure 6, where the coefficient of determination (R^2^) between the model-predicted average backfat thickness and the manually measured fat thicknesses at these five points is 0.7137.

## 4. Discussion

In this study, our objective was to automate the determination of pig carcass traits on a slaughter line using an ordinary camera. We employed a deep learning approach to automate the determination of the carcass length and backfat thickness. To achieve these objectives, we trained and validated the capability of our deep learning model by capturing images in a real slaughterhouse environment, following the operations of the automated slaughter line.

Carcass grading in pigs is now characterized by several non-destructive techniques. Over the past decades, numerous studies have explored the use of CT in meat production and animal research, which remains the most promising technique for determining lean, fat, and bone fractions, despite safety concerns [45,46]. Dual-energy X-ray Absorptiometry (DXA) relies on differential X-ray absorption by various tissues and offers advantages over CT in terms of instrument and installation costs [47]. However, DXA systems often require significant space and must be housed in lead-lined rooms for staff safety [47]. MRI technology faces similar challenges [17]. Ultrasound imaging is widely used in pigs as a non-destructive diagnostic tool, demonstrating good accuracy. Methods for backfat determination based on ultrasound imaging have also shown promising results, achieving a coefficient of determination R^2^ of 0.58 [48]. However, it is primarily used for live animals and is not suitable for automated slaughter line environments. With the emergence of low-cost high-definition cameras, we explored their application in automated slaughterhouse environments. The results show that our proposed method can provide significant assistance in grading pig carcasses.

In this study, we attempted to explore the effect of the camera based on different positions of the pig carcass on the results of automatic determination. The results were consistent with our conventional understanding; when the camera was positioned at the center of the carcass, the effect was better, which provides some reference for real application to the assembly line for automatic determination. When analyzing the errors that occurred during image acquisition, the images with the highest errors all showed different degrees of shooting angles, such as top view. After excluding the influence of human operation, it was found that this was due to the different lengths of pig carcasses after the position of the camera had been fixed. Therefore, it is important to note that the proposed method, when practiced in an automated slaughtering assembly line, should pay attention to the relative positions of the camera and pig carcasses, which may need to be refined by the corresponding engineering design experts.

We also compared the manual determination method with our proposed automated determination method, using the carcass phenotype of the subject as an example. The manual determination takes approximately 30 s, whereas the automated method takes only 2 s, with most of the time spent on manually acquiring the images. It then takes only a few milliseconds to produce results after inputting them into the model. It can be assumed that image acquisition time will be shorter and the efficiency faster when applied to automated slaughtering lines. Secondly, our proposed automated determination method maintains a consistent standard when assessing a large number of subjects, unlike manual methods, which are influenced by the assessment duration and subjective judgments. Our work highlights the efficacy of deep learning methods in automated pig carcass phenotyping, suggesting their potential utility in automating the determination of other phenotypic pig traits, thereby laying a solid foundation for pig breeding.

In this study, our deep learning-based automated determination method has proven effective in practically assessing carcass traits. However, the backfat segmentation dataset used has a limited sample size and lacks direct comparison with the five backfat positions used in manual determination. As a next step, we plan to enhance our method on a larger scale with more refined data. Acquiring carcass trait phenotypes can be achieved by simply installing low-cost high-definition cameras at specific locations in slaughterhouses. The primary aim of our proposed method is to enhance breeding efforts. Therefore, we intend to utilize this method for phenotype collection, simultaneously obtaining corresponding individual genotypes. This will enable a genome-wide association analysis to identify trait genes, further validating our method at another level.

## 5. Conclusions

In this study, we simulated an automated slaughter line within a real abattoir environment to acquire complete carcass and backfat images. We trained a deep learning-based automatic determination model to assess the carcass length and backfat thickness automatically. The feasibility of our models was tested by comparing their determinations with manually determined actual data, yielding R^2^ values of 0.9164, 0.9325, and 0.7137, respectively. Phenotypic determinations form the basis of breeding efforts, and leveraging big data enhances the accuracy of genetic evaluations. Our discussed methodology enables the acquisition of large quantities of phenotypic data for breeding purposes and provides technical support for the advancement of carcass grading techniques.

## Figures and Tables

**Table 1:** Different object detection models and comparison results.

Models	Class ^1^	Mean Average Precision	Parameters (M) ^2^	FLOPs(G) ^3^
SSD	Pubis	97.0	24.15	137.75
	The first rib	94.5		
	The first cervical vertebra	98.8		
	Internal reference	99.1		
Faster R-CNN	Pubis	58.5	41.14	78.13
	The first rib	37.5		
	The first cervical vertebra	25.0		
	Internal reference	52.6		
YOLOV5n	Pubis	96.3	1.76	4.2
	The first rib	97.9		
	The first cervical vertebra	99.0		
	Internal reference	99.4		
YOLOV8n	Pubis	98.2	3.01	8.1
	The first rib	99.4		
	The first cervical vertebra	99.1		
	Internal reference	99.5		

^1^ Detection classes in the constructed dataset; ^2^ parameters: the size of the number of parameters is an important evaluation metric for lightweight models; M: million; ^3^ FLOPs: the number of floating-point operations, which can be used to measure the algorithm/model complexity.

**Table 2:** Segmentation performance of the backfat region for each image segmentation model.

Model Name	Mean Accuracy	Mean IoU	Precision	Recall	F1-Score
YOLOV8n-seg	97.23	89.10	96.86	97.23	97.03
U-Net	96.83	86.37	95.70	96.83	96.22
PSP-Net	93.29	79.70	95.04	93.29	94.09
Deeplabv3	96.52	84.81	95.02	96.52	95.73
YOLOV5n-seg	97.02	88.97	96.15	97.08	96.89
FCN	92.02	69.65	89.56	92.02	90.45

## Data Availability

The raw data supporting the conclusions of this article will be made available by the authors upon request.
